# Non‐invasive cardiac power measurements in decompensated heart failure in elderly adults: A prospective proof‐of‐concept study

**DOI:** 10.14814/phy2.70795

**Published:** 2026-03-22

**Authors:** Ingrid Yttervoll, Andreas Østvik, Petter Aadahl, Rune Mo, Bjørnar Grenne, Idar Kirkeby‐Garstad

**Affiliations:** ^1^ Department of Circulation and Medical Imaging, Faculty of Medicine and Health Sciences Norwegian University of Science and Technology Trondheim Norway; ^2^ Medical Student Research Programme, Faculty of Medicine and Health Sciences Norwegian University of Science and Technology Trondheim Norway; ^3^ Department of Cardiology St. Olavs University Hospital Trondheim Norway; ^4^ Medical Image Analysis Health Research, SINTEF Digital Trondheim Norway; ^5^ Department of Anaesthesia and Intensive Care St. Olavs University Hospital Trondheim Norway

**Keywords:** cardiac power, haemodynamics, heart failure, oscillatory power fraction

## Abstract

In this proof‐of‐concept study, we evaluated the feasibility of non‐invasive estimation of cardiac power metrics—total power, steady power, oscillatory power, and oscillatory power fraction—and compared the prognostic and diagnostic value with established echocardiographic metrics. We prospectively included 29 patients (mean age 76 ± 13 years, 24% women) hospitalized with decompensated heart failure. Left ventricular outflow tract flow waveforms were derived from Doppler echocardiography and synchronized with a continuous arterial pressure waveform from a finger‐volume‐clamp device (INL382, Finapres Medical Systems B.V., Amsterdam, Netherlands). Total power was computed as the integral of the instantaneous pressure‐flow product per second, steady power as mean arterial pressure multiplied by mean flow, and oscillatory power as their difference. All measures were calculated from the same consecutive heartbeats covering three respiratory cycles. The feasibility of obtaining cardiac power metrics was 91%. Total, steady, and oscillatory power predicted short‐term all‐cause mortality (log rank *p* < 0.015), whereas left ventricular ejection fraction, global longitudinal strain, and myocardial work indices did not. These findings suggest that cardiac power metrics may be useful in risk stratification and warrant validation in larger cohorts.

## 1 INTRODUCTION

Heart failure is a global pandemic affecting over 64 million people worldwide in 2017 and is projected to increase due to an aging population (James et al., 2018; Savarese et al., 2023). The heart failure syndrome is divided into subgroups based on left ventricular ejection fraction (LVEF), which defines guideline‐directed treatment decisions (Bozkurt et al., 2021). LVEF has both strengths and limitations. It is easily obtained non‐invasively with echocardiography, and its ability to predict prognosis is well established (Curtis et al., 2003; Solomon et al., 2005). On the other hand, LVEF‐based subgroups are heterogeneous in terms of both pathophysiology, prognosis, and response to treatment (Cohen et al., 2020; Heidenreich et al., 2022; Konstam & Abboud, 2017; Solomon et al., 2005). This heterogeneity is partially due to important limitations of LVEF, such as its geometric assumptions and load dependency (Jain & Borlaug, 2020; Konstam & Abboud, 2017; Monge García et al., 2019). Left ventricular (LV) global longitudinal strain (GLS) has emerged as an adjunct method for evaluating LV function, but it is also load dependent (Lang et al., 2015). To address methodological challenges, recent research has emphasized multimodal approaches that combine clinical parameters and biomarkers with easily obtainable echocardiographic measures to provide a more comprehensive assessment of haemodynamics in heart failure (Cikes & Solomon, 2016). A prominent example is the myocardial work indices (global work index, global constructive work, global wasted work, and global work efficiency), which are derived by combining strain with estimated systolic pressure waveforms (Russell et al., 2012).

Cardiac power metrics (total power, steady power, oscillatory power, and oscillatory power fraction) derived from cardiac power have been proposed as clinically valuable in haemodynamically unstable patients (Rimehaug et al., 2013). Cardiac power refers to the hydraulic power burst generated by the heart and transferred to the aorta during systole. Instantaneous cardiac power is calculated by multiplying instantaneous blood pressure by instantaneous flow. The cardiac power peak (maximal power) depends mainly on left ventricular filling and contractility (Kass & Beyar, 1991). The integral of cardiac power for one cardiac cycle is equal to stroke work (Westerhof et al., 2018), representing the total energy transferred from the left ventricle to the aorta in that cycle.

Total power is the energy transferred from the heart to the aorta in any natural number of cardiac cycles divided by time, reflecting the rate at which this energy is delivered, thereby linking ventricular work to circulatory load and metabolic demand. The total power can be separated into two components: steady power, which represents the component driving the blood flow forward, and oscillatory power, which reflects the energy associated with arterial pulsations. A parameter related to steady power, cardiac power output, has been shown to be a strong predictor of mortality in cardiogenic shock (Fincke et al., 2004), whereas the oscillatory power fraction (oscillatory power relative to total power) has been proposed as an indicator of ventriculo‐arterial coupling (Cholley & Le Gall, 2016; Tannvik et al., 2019).

Traditionally, these cardiac power metrics have been estimated invasively through heart catheterisation. More recently, less invasive methods have combined echocardiographic flow estimation with invasive radial pressure measurements (Rimehaug et al., 2016; Tannvik et al., 2019). In this study, we advance the methodology by deriving both pressure and flow non‐invasively and evaluate its feasibility in patients with decompensated heart failure. We further compare the diagnostic, prognostic, and treatment‐response value to established echocardiographic measures, including LVEF, GLS, and myocardial work indices.

## 2 METHODS

In this proof‐of‐concept study, we prospectively included patients admitted to St. Olav's University Hospital between January and June 2023 with decompensated heart failure. Eligibility was based on the treating physician's assessment at inclusion, identifying patients who were clinically decompensated and not yet receiving optimal treatment. A random subset of patients underwent a follow‐up examination after hospital discharge a mean of 60 ± 27 days after inclusion. We collected patient demographics, medical histories, and all‐cause mortality data from patients' medical records.

### 2.1 Pathophysiology

Patients were categorized into aetiologic groups with a common pathophysiology. The patient's heart failure aetiology was determined by the treating physician and by an independent, blinded expert cardiologist (BG) based on echocardiographic findings and medical history. For pathophysiology‐based analyses, patients with ischaemic cardiomyopathy or dilated cardiomyopathy were grouped as “reduced systolic function”, and patients with suspected amyloidosis were grouped as “reduced filling function”. The grouping was guided by the American Heart Association Scientific Statement on infiltrative cardiomyopathies and recent state‐of‐the‐art reviews on dilated and ischaemic cardiomyopathies (Heymans et al., 2023; Kottam et al., 2023; Pastena et al., 2024).

### 2.2 Echocardiographic acquisition and measurement

Echocardiographic examinations followed American Society of Echocardiography and European Association of Cardiovascular Imaging recommendations (Lang et al., 2015). All scans were acquired on a GE HealthCare Vivid E95 system (GE HealthCare, Horten, Norway) by one of two level III echocardiographers (BG and RM). Heart rate was derived from the three‐lead electrocardiogram (ECG) that all patients wore during the examination. Left ventricular outflow tract (LVOT) flow was assessed with pulsed‐wave Doppler just proximal to the aortic valve. LVOT diameter was measured in the parasternal long‐axis view and averaged over three cardiac cycles. Stroke volume was calculated as LVOT cross‐sectional area × LVOT velocity–time integral. LVEF was measured by the biplane Simpson method, GLS with the 2D Strain tool, and myocardial work indices with the myocardial work module, all in EchoPAC (GE HealthCare, version 206). The global work index corresponds to the area enclosed by the pressure–strain loop (Russell et al., 2012). Global constructive work represents myocardial shortening during systole and lengthening during diastole. Conversely, global wasted work reflects shortening during diastole and lengthening during systole. Global work efficiency was calculated as the ratio of global constructive work to the sum of global constructive and wasted work. All measurements were performed by an expert cardiologist (BG), with cardiac event timing (mitral/aortic valve opening and closure) for myocardial work annotated by a second observer (IY) under supervision (BG).

### 2.3 Cardiac power metrics calculation

The method for calculating cardiac power metrics has been validated in previous invasive and experimental animal studies (Rimehaug et al., 2013; Rimehaug et al., 2016; Tannvik et al., 2019). In this study, we implemented the approach using an in‐house application that imported Doppler flow‐velocity waveforms, LVOT diameter measurements, and ECG signals from the echocardiographic examination, together with arterial pressure waveforms from a Finapres finger‐cuff device (INL382, Finapres Medical Systems B.V., Amsterdam, Netherlands).

**FIGURE 1:**
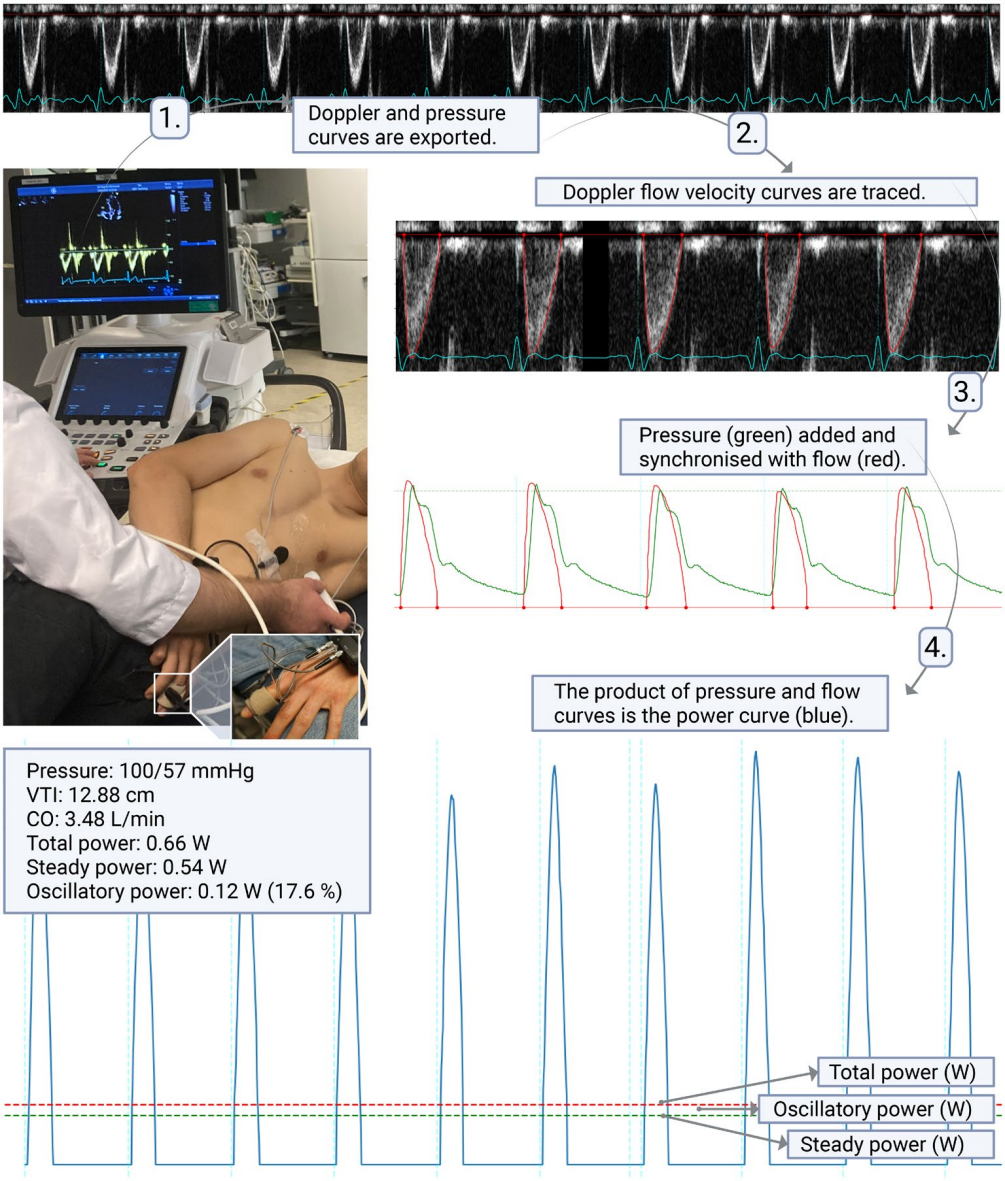
Step‐by‐step workflow for calculating cardiac power metrics from pulsed‐wave Doppler and peripheral blood pressure data. LVOT, left ventricular outflow tract. Created in https://BioRender.com.

The Doppler flow‐velocity waveform was manually traced by one observer (IY) under continuous supervision (BG) and converted to volumetric flow. Flow and pressure signals were then synchronized to the same heartbeats by manually aligning the systolic upstroke of the pressure waveform with the corresponding upstroke of the (inverted) flow waveform, maximizing overlap of the upstroke segments across beats (Figure 1 step 3). Accurate synchronization was facilitated by a live annotation inserted into the exported pressure recording, marking the exact start and end of the echocardiographic flow acquisition. The instantaneous power waveform was defined as the product of flow and pressure.

Total power was calculated as the time integral of the instantaneous power waveform divided by the sampling interval in seconds. Steady power was calculated as the product of the mean arterial pressure and the mean volumetric flow. Both total and steady power were estimated over the same consecutive heartbeats spanning approximately three respiratory cycles. Oscillatory power was defined as the difference between total and steady power, and the oscillatory power fraction as the ratio of oscillatory power to total power. Figure 1 illustrates the step‐by‐step workflow, and Table 1 summarizes the definitions and formulas for the cardiac power metrics.

**TABLE 1:** Cardiac power metrics.

Metric	Explanation	Formulas
Instantaneous power (W)	Hydraulic power (product of instantaneous pressure and flow) at a specific instant t.	$P\left(t\right)\;Q\left(t\right)$
Total power (W)	Average hydraulic energy per time T (including both pulsatile and steady component) delivered by the left ventricle to the arteries.	$\frac{1}{T}\int_{0}^{T}P\left(t\right)\;Q\left(t\right)\;dt$
Steady power (W)	Non‐pulsatile component of hydraulic power.	$\bar{P\left(t\right)}\cdot \bar{Q\left(t\right)}$
Oscillatory power (W)	Pulsatile power transferred to oscillations in the large arteries.	$\frac{1}{T}\int_{0}^{T}P\left(t\right)\;Q\left(t\right)\;dt-\bar{P\left(t\right)}\cdot \bar{Q\left(t\right)}$
Oscillatory power fraction (%)	Fraction of total power due to pulsatility.	$\left(1-\frac{\bar{P\left(t\right)}\cdot \bar{Q\left(t\right)}}{\frac{1}{T}\int_{0}^{T}P\left(t\right)\;Q\left(t\right)\;dt)}\right)\cdot 100\%$

*Note*: *P*(t), instantaneous pressure (mmHg); *Q*(t), instantaneous volumetric flow rate (m^3^/s); *T*, duration of sampling interval (s).

### 2.4 Feasibility

Feasibility was defined as the proportion of patients in whom cardiac power metrics could be successfully calculated. Successful calculation required Doppler flow‐velocity waveforms of adequate quality and correctly acquired arterial pressure waveforms.

### 2.5 Statistics

We used *p* < 0.05 as the significance level. Continuous normally distributed data are displayed as mean ± SD, and non‐normally distributed data are presented as median with IQR. Normality was evaluated using QQ plots, histograms, and the Shapiro–Wilk test. Differences between groups were analyzed using the Student t‐test, Welch test, or Mann–Whitney *U* test, as appropriate. Homogeneity of variance was ensured with Levene's test. We used linear regression to assess the correlation between cardiac power metrics and conventional echocardiographic measurements (GLS, LVEF and myocardial work indices). We used cardiac power metrics as predictors and conventional echocardiographic measurements as outcomes. Linear regressions are reported with robust standard errors, and outcomes are log or logit‐transformed as appropriate to meet assumptions. The mortality analysis is presented using Kaplan–Meier curves with 95% confidence intervals calculated using Greenwood's formula and a log‐rank *p*‐value, as well as a weighted average of time‐varying hazard ratios calculated with Cox regression. Time‐to‐event was defined as the time from inclusion to either death or censoring at the study's end date (approximately 23 months after end of inclusion). The outcome was all‐cause mortality. We constructed receiver operating characteristic curves for each parameter. We selected the optimal cut‐off value by choosing the threshold that maximized the Youden index. To compare patients' values during admission with after discharge, we used a paired t‐test for normally distributed data and the Wilcoxon signed‐rank test for non‐normally distributed data.

### 2.6 Ethical approval

The study was approved (480738) by the Regional Committee for Medical Research Ethics. We conducted the study in accordance with the principles outlined in the Declaration of Helsinki. Written informed consent was obtained from all patients.

## 3 RESULTS

The study population comprised 29 patients (mean age 76 ± 11 years, 24% women). Three patients were excluded due to poor image quality, pressure acquisition error, and untraceable VTI measurement, respectively. The feasibility of cardiac power metrics was 91% (29/32). Demographic characteristics and cardiovascular risk factors are summarized in Table 2, and left ventricular function and mechanics variables in Table 3. We were unable to obtain GLS and myocardial work indices from four (14%) of the included patients due to insufficient image quality for strain analysis.

**TABLE 2:** Demographic data and cardiovascular risk factors (*n* = 29).

Characteristic	Value
Age, years, mean (SD)	76 (11)
Weight, kg, mean (SD)	81 (16)
Body mass index, kg/m^2^, mean (SD)^a^	26 (5)
Velocity‐time integral (cm), mean (SD)	13 (5)
Stroke volume (mL), mean (SD)	60 (19)
Systolic pressure (mmHg), mean (SD)	114 (22)
Diastolic pressure (mmHg), mean (SD)	49 (10)
Heart rate (bpm), median (IQR)	78 (25)
Mitral regurgitation, *n* (%)	17 (59)
Aorta regurgitation, *n* (%)	6 (21)
Aorta stenosis, *n* (%)	4 (14)
Hypertension, *n* (%)	16 (55)
Kidney failure, *n* (%)	2 (7)
Diabetes, *n* (%)	12 (41)
Hypercholesterolemia, *n* (%)	4 (14)
Previous myocardial infarction, *n* (%)	10 (34)
Previous percutaneous coronary intervention, *n* (%)	9 (31)
Previous bypass intervention, *n* (%)	5 (17)
Coronary artery disease, *n* (%)	8 (28)
Atrial fibrillation, *n* (%)	9 (31)
Implantable cardioverter‐defibrillator, *n* (%)	2 (7)
Pacemaker, *n* (%)	2 (7)
Chronic obstructive pulmonary disease, *n* (%)	4 (14)
Obstructive sleep apnea syndrome, *n* (%)	3 (10)

Abbreviation: SD, standard deviation.

^a^ Data available for 25 patients.

**TABLE 3:** Left ventricular function and mechanics (*n* = 29).

Parameter	Value
Total power (W), mean (SD)	0.89 (0.38)
Steady power (W), mean (SD)	0.66 (0.26)
Oscillatory power (W), median (IQR)	0.21 (0.11)
Oscillatory power fraction (%), mean (SD)	25 (5)
Ejection fraction (%), mean (SD)	34 (12)
Global longitudinal strain (%)^a^, median (IQR)	−7.7 (4.0)
Global work index (mmHg%)^a^, median (IQR)	708 (442)
Global constructive work (mmHg%)^a^, median (IQR)	944 (349)
Global wasted work (mmHg%)^a^, median (IQR)	156 (125)
Global work efficiency^a^, median (IQR)	0.86 (0.11)

Abbreviations: IQR, interquartile range; LVOT, left ventricular outflow tract; SD, standard deviation.

^a^ Data available for 25 patients.

Nine patients had a mean systolic blood pressure > 130 mmHg during examination and a mean oscillatory power fraction of 29 ± 5%. The remaining 20 patients had a significantly lower mean oscillatory power fraction of 23 ± 4% (*p* = 0.005).

### 3.1 Cardiac power in relation to conventional echocardiographic measurements

All cardiac power metrics were significantly correlated with LVEF (all *p* < 0.001). Only total power and oscillatory power fraction showed a significant correlation with GLS (all *p* < 0.028). Among cardiac power metrics, oscillatory power fraction demonstrated the strongest association with both LVEF (*R* ^2^ = 0.41), global work index (*R* ^2^ = 0.55), and GLS (*R* ^2^ = 0.59). All correlations between cardiac power metrics and conventional echocardiographic measurements are presented in Table S1, and the relation with LVEF in Figure S1.

### 3.2 Cardiac power in different heart failure pathophysiologies

**FIGURE 2:**
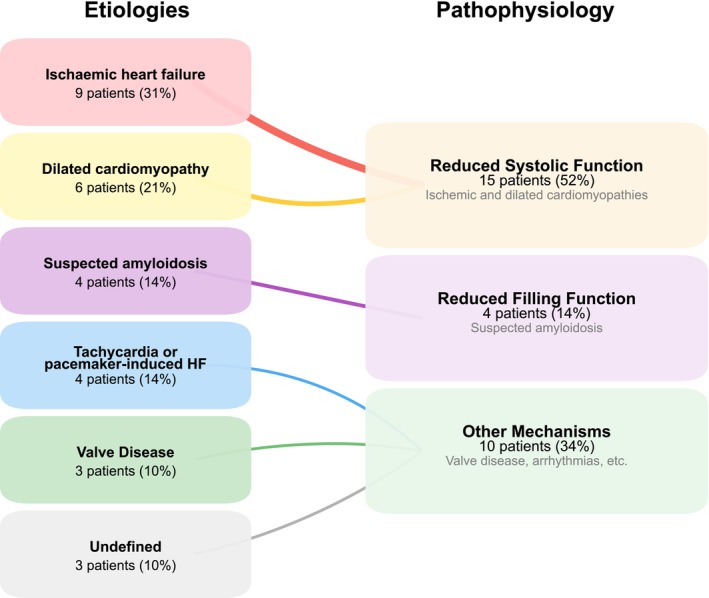
Heart failure aetiologies matched with their underlying predominant pathophysiology.

**FIGURE 3:**
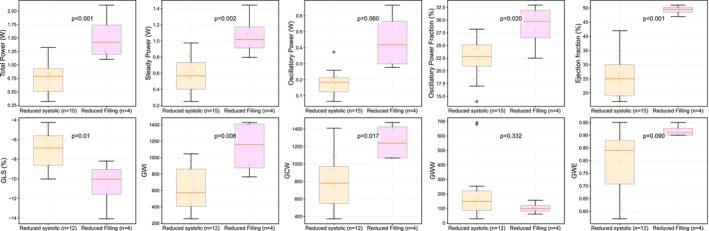
Boxes show the interquartile range, with whiskers extending to the most extreme points within 1.5 times the range. Points beyond are outliers. The horizontal line is the median. Jittered dots show individual patients. *p*‐values are for pathophysiology‐group differences detected by each parameter. GCW, global constructive work; GLS, global longitudinal strain; GWI, global work index; GWW, global wasted work; GWE, global work efficiency.

The aetiologies and predominant pathophysiology of the patients are shown in Figure 2. Most patients (52%) had a predominant pathophysiology of reduced systolic function, while 14% had a predominant pathophysiology of reduced filling function. Patients with reduced systolic function had significantly lower total power, steady power, oscillatory power fraction, LVEF, GLS, global work index, and global constructive work (all *p* < 0.05) compared to patients with reduced filling function. In contrast, oscillatory power, global wasted work, and efficiency showed no significant differences (Figure 3). In Figure S2, all pathophysiological subgroups are visualized.

### 3.3 Mortality

**FIGURE 4:**
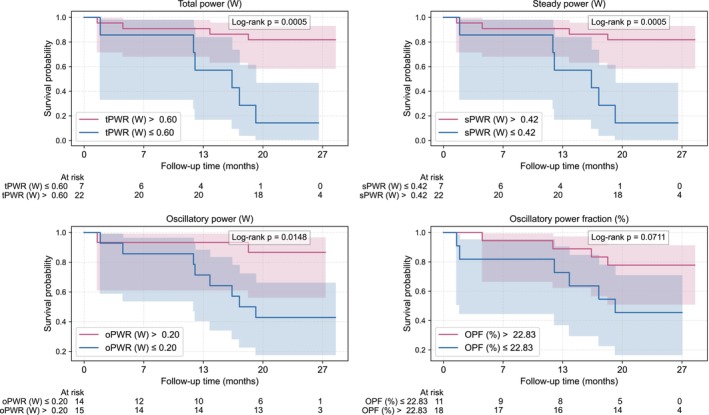
Kaplan–Meier survival curves. The shaded area represents the 95% confidence intervals. OPF, oscillatory power fraction; oPWR, oscillatory power; sPWR, steady power; tPWR, total power.

**FIGURE 5:**
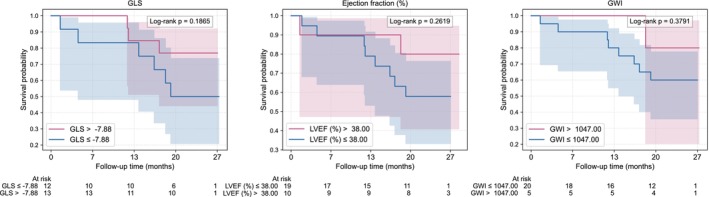
Kaplan–Meier survival curves. The shaded area represents the 95% confidence intervals. GLS, global longitudinal strain; GWI, global work index; LVEF, left ventricular ejection fraction.

Follow‐up was complete for all patients. Among survivors, the median follow‐up was 26 months (IQR 2.2 months). By the end of follow‐up, 10 patients (34%) had died. Using AUC‐derived cutoffs, all cardiac power metrics significantly separated survival curves (log‐rank *p* < 0.015) except for oscillatory power fraction (log‐rank *p* = 0.071) (Figure 4). In contrast, LVEF, GLS, and the global work index did not discriminate survival (all *p* > 0.187) (Figure 5). The remaining myocardial work indices all yielded significant differences and are presented in Figure S3. Oscillatory, total and steady power demonstrated significant prognostic performance, with a time‐weighted average hazard ratio of 0.33 (CI 0.11–0.97, *p* = 0.044), 0.35 (CI 0.14–0.86, *p* = 0.022) and 0.37 (CI 0.16–0.88, *p* = 0.024) respectively. This corresponds to a 67% lower hazard of death per 0.13 W increase in oscillatory power, a 65% lower hazard of death per 0.38 W increase in total power and a 63% lower hazard of death per 0.26 W increase in steady power. A summary of hazard ratios, p‐values, and AUCs for all parameters is provided in Table 4 and Table S2.

**TABLE 4:** Summary of Hazard ratios and *p*‐values.

Parameter	Mean (SD)	Hazard ratio per 1 SD (95% CI)	Hazard ratio *p*‐value	AUC
Total power (W)	0.89 (0.38)	0.35 (0.14–0.86)	0.022	0.76
Steady power (W)	0.66 (0.26)	0.37 (0.16–0.88)	0.024	0.78
Oscillatory power (W)	0.23 (0.13)	0.33 (0.11–0.97)	0.044	0.76
Oscillatory power fraction (%)	25.1 (5.2)	0.67 (0.33–1.34)	0.256	0.65
Left ventricular ejection fraction (%)	34 (12)	0.92 (0.49–1.74)	0.794	0.52
Global longitudinal strain (%)	−8.5 (3.7)	0.95 (0.51–1.77)	0.879	0.56
Global work index (mmHg%)	805 (487)	1.05 (0.56–1.97)	0.871	0.51

Abbreviation: AUC, area under curve.

### 3.4 Changes after treatment

When we compared a subset of patients pairwise from admission to after discharge from the hospital (*n* = 10), we observed no significant change in any cardiac power metrics, LVEF, GLS, or myocardial work indices (all *p* > 0.2).

## 4 DISCUSSION

Our main finding in this exploratory proof‐of‐concept study is that cardiac power metrics have high feasibility in decompensated heart failure when assessed non‐invasively. Moreover, these metrics show promise in predicting short‐term all‐cause mortality as well as identifying underlying heart failure pathophysiologies.

### 4.1 Cardiac power metrics related to reference values

Overall, the mean total, steady, and oscillatory power in patients with decompensated heart failure was lower than that reported in healthy participants by Nichols et al. (Nichols et al., 1986). Nichols et al. measured cardiac power metrics using an invasive catheter in the ascending aorta in 11 healthy participants and reported mean values of 1.58 W for total power, 1.37 W for steady power, and 0.21 W for oscillatory power. Conceptually, it is not surprising that patients with decompensated heart failure exhibit lower cardiac energy transfer than healthy individuals. We nevertheless caution that absolute values are not directly interchangeable to our reported findings owing to substantial methodological differences between the studies.

Patients with decompensated heart failure exhibited a higher mean oscillatory power fraction of 25 ± 5% than Nichols' reported mean of 13 ± 2.7%. Nichols et al. further found that among the hypertensive patients (*n* = 11) the oscillatory power fraction significantly increased by 38% to mean 18 ± 3.6%. These findings are consistent with a relatively greater proportion of energy being transferred to oscillatory components in patients with decompensated heart failure compared to healthy individuals. Part of this elevation may be attributed to hypertension, as higher oscillatory power fractions are associated with elevated blood pressure (Tannvik et al., 2019; Westerhof et al., 2018). During the examination, nine patients had systolic blood pressure ≥ 130 mmHg and they exhibited higher oscillatory power fraction. We used a threshold of 130 mmHg to define hypertension, rather than guideline‐recommended 140 mmHg (ADInstruments NZ Limited, 2022; McEvoy et al., 2024) because the Finapres device documentation reports an underestimation of brachial pressure by about 10 mmHg (ADInstruments NZ Limited, 2022). Additionally, 55% of the patients had a documented history of hypertension in their medical records. Taken together, the observed hypertension likely contributes to the elevated oscillatory power fraction through mechanisms like arterial stiffening and increased wave reflections; however, more research on normative non‐invasive human reference ranges for cardiac power metrics is needed to make accurate comparisons.

### 4.2 Cardiac power in different heart failure patophysiologies

Cardiac power metrics differentiated between underlying heart failure pathophysiologies. Notably, the group with suspected cardiac amyloidosis, characterized by reduced filling function, exhibited a higher oscillatory power fraction compared to those with reduced systolic function. A previous study by Tannvik et al. has reported oscillatory power fraction of respectively 23 ± 6% and 16 ± 4% in 28 patients before and after undergoing elective coronary artery bypass grafting (Tannvik et al., 2019). Our average oscillatory power fraction exceeds their findings. Tannvik proposed that oscillatory power fraction may serve as a marker of ventriculo‐arterial uncoupling, reflecting the relationship between dynamic impedance and available contractility. The elevated oscillatory power fraction observed in the amyloidosis group may indicate a greater degree of uncoupling relative to patients with reduced systolic function. This interpretation is further supported by the documented increases in ventricular and arterial stiffness in patients with amyloidosis (Korela et al., 2025).

All cardiac power metrics except for oscillatory power were significantly higher in the group with reduced filling function compared to the group with reduced systolic function. Effective treatment of cardiac amyloidosis depends on early diagnosis, and there is a growing need for non‐invasive diagnostic screening tools (Kittleson et al., 2023). These findings suggest that in a population of decompensated heart failure patients, cardiac power metrics may be an additional clinical clue in identifying potential cases of amyloidosis. It is important to note that due to the limited sample size, larger studies are needed to confirm this hypothesis.

### 4.3 Mortality

Oscillatory total and steady power significantly predicted short‐term mortality in our cohort. These findings align with previous studies that identified reduced cardiac power output, a corresponding parameter, as a robust predictor of mortality in heart failure patients (Fincke et al., 2004; Lang et al., 2009; Yildiz et al., 2017). Cardiac power output differs from steady power in that it is not calculated over multiple synchronized heartbeats but from measurements of peripheral arterial blood pressure times cardiac output obtained at different time points. In contrast to cardiac power metrics, LVEF, GLS, and global work index did not predict mortality in our study. These findings support the potential clinical value of cardiac power metrics for risk stratification in patients with decompensated heart failure.

Interestingly, a decrease in global wasted work and an increase in global work efficiency were significantly associated with lower mortality. Yedidya et al. (2021) found similar results in a population with secondary mitral regurgitation and suggested that this could be explained by the LV emptying into a low‐pressure atrium. In our cohort, 59% of the patients had mitral regurgitation, which may explain the results.

### 4.4 Changes after treatment

There were no significant differences in any cardiac power metrics between measurements taken during hospital admission and those obtained after discharge. We initially hypothesized that optimizing heart failure treatment during admission would improve ventriculo‐arterial coupling, leading to a reduced oscillatory power fraction at follow‐up. However, this was not observed. The lack of reduction in oscillatory power fraction could be due to patients not actually experiencing improved coupling at the follow‐up, but this remains unclear since we did not have access to gold‐standard invasive measurements of ventriculo‐arterial coupling. Potential existing changes could also have been obscured by the small sample size and heterogeneity among the included patients.

### 4.5 Limitations and strengths

Because this was an exploratory, proof‐of‐concept study, we included all patients with decompensated heart failure regardless of aetiology. This broad inclusion, together with the limited sample size, precludes in‐depth analyses of how aetiological and pathophysiological subtypes differentially affect non‐invasive cardiac power metrics. Nevertheless, this approach offers a first overview of the method's overall clinical performance in an unselected heart failure population.

A clear definition of normal resting cardiac power is not well established, neither for our non‐invasive approach nor for more invasive measurements. This uncertainty constrains absolute interpretation and external validation of the reported values.

As with other low‐invasive cardiac power estimations, flow and pressure were acquired at different vascular sites: forward flow across the aortic valve by Doppler, and arterial pressure peripherally at the finger. Because systolic peak waveform morphology differs between the central aorta and peripheral arteries (owing to vessel caliber, wall stiffness, and wave reflections), and these differences vary across individuals, calculating instantaneous power and its components introduces uncertainty. A transfer‐function recalculation from finger to aortic pressure would require an additional continuous recording at another site, which was not feasible here. Absolute cardiac power metrics may therefore be overestimated, due to higher peripheral systolic pressure. By contrast, the oscillatory power fraction, being a ratio, may be less sensitive to site‐specific amplitude differences, although waveform morphology could still influence this metric. Accordingly, our estimates should be regarded as approximate yet physiologically plausible.

The method is fully non‐invasive and showed high feasibility. Importantly, left ventricular flow and arterial pressure were synchronized and derived from the same series of consecutive heartbeats spanning multiple respiratory cycles. This captures both respiratory and beat‐to‐beat variability in pressure and flow and improves the representation of the average pressurized volume transferred from the heart to the vasculature over the analyzed interval.

### 4.6 Future work

Future work should elucidate patterns in cardiac power metrics across aetiology‐defined heart failure subgroups and healthy controls, using adequately powered cohorts. Aetiologies of particular interest include ischaemic heart failure, dilated cardiomyopathy, and biopsy‐ or imaging‐confirmed cardiac amyloidosis. In addition, the temporal dynamics of cardiac power in relation to the varying degrees of ventriculo–arterial coupling warrant further investigation.

## 5 CONCLUSION

Cardiac power metrics show high feasibility when measured non‐invasively in decompensated heart failure patients. Cardiac power metrics also show promise in predicting short‐term all‐cause mortality, as well as in differentiating between heart failure pathophysiologies. These findings, although hypothesis‐generating given the small sample size, support further validation in larger cohorts.

## FUNDING INFORMATION

This work was financially supported by internal research funds from St. Olavs Hospital and the Norwegian University of Science and Technology.

## CONFLICT OF INTEREST STATEMENT

The authors declare no potential conflict of interest.

## Supporting information


Data S1.

## Data Availability

The data that support the findings of this study are available on request from the corresponding author. The data are not publicly available due to privacy or ethical restrictions.
