# Supplementary material for: Non‐invasive cardiac power measurements in decompensated heart failure in elderly adults: A prospective proof‐of‐concept study
Source: Physiol Rep. 2026 Mar 22;14(6):e70795. doi: 10.14814/phy2.70795 (PMC13097327; doi:10.14814/phy2.70795)
Supplement: Supplementary file 1 — Data S1. [file PHY2-14-e70795-s001.pdf]

□

# APPENDIX

## SUPPLEMENTAL TABLES

Supplementary tables 1 and 2.

**TABLE 1** Linear regression between cardiac power metrics and echocardiographic measurements

| Outcomes | Predictors | | | |
|---|---|---|---|---|
| | Total power (W) | Steady power (W) | Oscillatory power (W) | Oscillatory power fraction (%) |
| LVEF (%) | $\beta = 17.1^c$, $R^2 = 0.27$ | $\beta = 21.2^c$, $R^2 = 0.20$ | $\beta = 59.7^c$, $R^2 = 0.40$ | $\beta = 1.5^c$, $R^2 = 0.41$ |
| GLS (%) | $\beta = -3.6^b$, $R^2 = 0.15$ | $\beta = -3.6^a$, $R^2 = 0.07$ | $\beta = -15.8^a$, $R^2 = 0.34$ | $\beta = -0.6^c$, $R^2 = 0.59$ |
| GWI (mmHg%) | $\beta = 630^c$, $R^2 = 0.25$ | $\beta = 727^c$, $R^2 = 0.16$ | $\beta = 2393^b$, $R^2 = 0.43$ | $\beta = 71.3^b$, $R^2 = 0.55$ |
| GCW (mmHg%) | $\beta = 604^b$, $R^2 = 0.23$ | $\beta = 699^b$, $R^2 = 0.15$ | $\beta = 2280^b$, $R^2 = 0.39$ | $\beta = 70.7^c$, $R^2 = 0.53$ |
| GWW (mmHg%) | $\beta = -0.1^a$, $R^2 = 0.00$ | $\beta = -0.1^a$, $R^2 = 0.00$ | $\beta = -0.5^a$, $R^2 = 0.01$ | $\beta = -0.0^a$, $R^2 = 0.02$ |
| GWE | $\beta = 0.7^a$, $R^2 = 0.11$ | $\beta = 0.9^a$, $R^2 = 0.07$ | $\beta = 2.6^a$, $R^2 = 0.17$ | $\beta = 0.1^b$, $R^2 = 0.22$ |

**TABLE 1** Superscripts indicate significance: $^a$ $P > 0.05$, $^b$ $P < 0.05$, $^c$ $P < 0.001$. GLS, global longitudinal strain; GWI, global work index; GCW, global constructive work; GWW, global wasted work; GWE, global work efficiency.

**TABLE 2** Hazard ratio, AUC, and p-values

| Parameter | Mean (SD) | Hazard ratio per 1 SD (95% CI) | Hazard ratio *P*-value | AUC |
|---|---|---|---|---|
| Global constructive work (mmHg%) | 1016 (489) | 0.99 (0.51–1.91) | $P = 0.968$ | 0.53 |
| Global wasted work (mmHg%) | 192 (172) | 0.81 (0.35–1.89) | $P = 0.627$ | 0.65 |
| Global work efficiency | 0.83 (0.12) | 1.31 (0.61–2.81) | $P = 0.494$ | 0.58 |

**TABLE 2** AUC, area under curve.

## SUPPLEMENTAL FIGURE

Supplemental figures 1, 2 and 3.

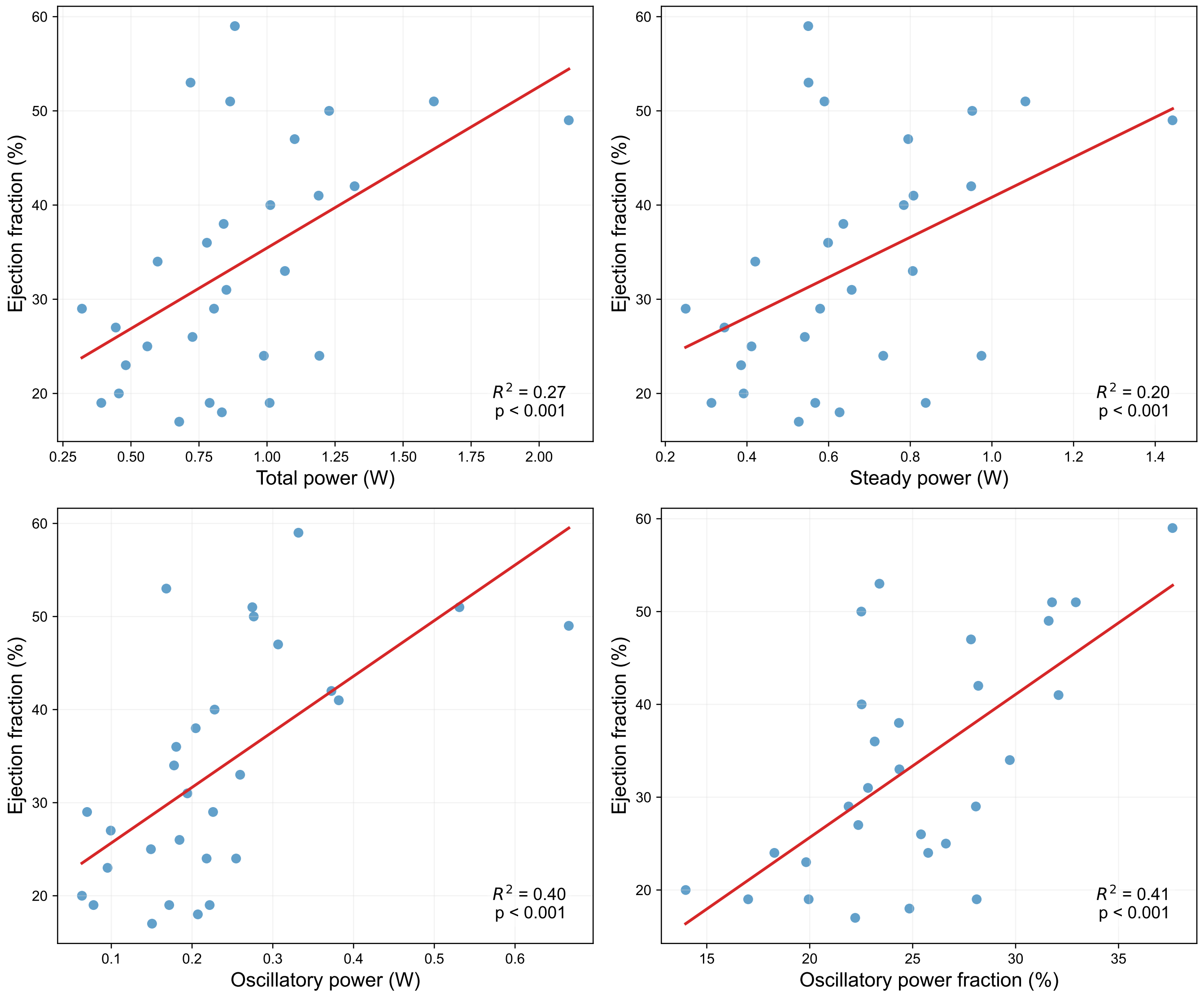


**FIGURE 1** Scatter plots showing the relationship between cardiac power metrics (x-axis) and left ventricular ejection fraction (y-axis) with linear regression lines in red. $R^2$ values and p-values are displayed for each group.

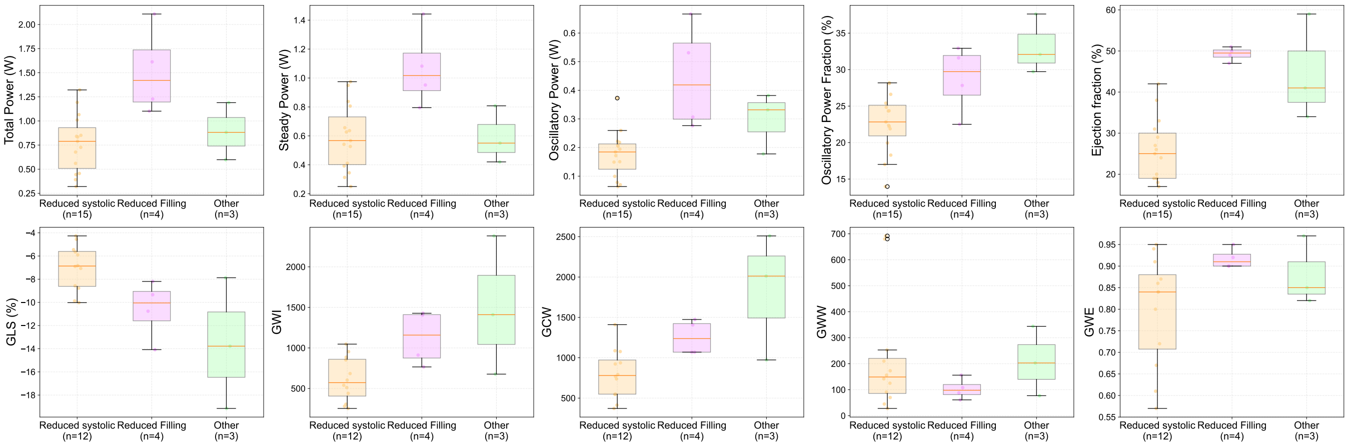


**FIGURE 2** Boxes show the interquartile range, with whiskers extending to the most extreme points within 1.5 times the range. Points beyond are outliers. The horizontal line is the median. Jittered dots show individual patients. GCW, global constructive work; GLS, global longitudinal strain; GWI, global work index; GWW, global wasted work; GWE, global work efficiency.

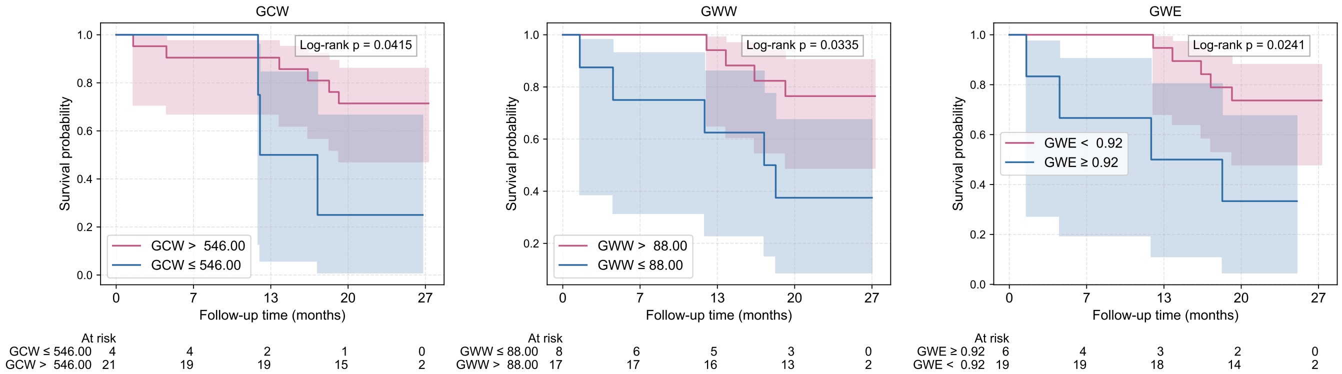


**FIGURE 3** Kaplan–Meier survival curves. The shaded area represents the 95% confidence intervals. GCW, global constructive work; GWW, global wasted work; GWE, global work efficiency.
